# Protracted Abstinence from Food

**Published:** 1873-08

**Authors:** A. Ford

**Affiliations:** Bowne, Kent Co., Mich.


ARTICLE IV.—*Protracted Abstinence from Food.* By A. FORD, M.D., of Bowne, Kent Co., Mich.

April 25, 1873, was called to see Mr. M., aged 74 years. Farmer; born in the Highlands of Scotland; five feet ten inches in height; quite fleshy.

Found him suffering from what appeared to be bilious colic; severe pain in the epigastric region, with constipation of the bowels. Relieved the pain with hypodermic injection of morphia, and then gave physic. Did not get an operation for three days, then used the syringe, which relieved him.

May 3, was called again. Symptoms the same, with the addition of excessive nausea and vomiting. The pain came on periodically, about eleven o'clock P. M. Through the day he was nearly free from pain.

Failing to relieve him, and being obliged to leave town for two days, sent for Dr. M. to see him for me. He gave him large doses of calomel. After some two days got another operation, with partial relief.

He had now become somewhat emaciated, and on a very careful examination, discovered a small tumor in the region of the pylorus. Dr. M. saw him with me again. Concluded that the tumor, though small, pressed upon, or involved the pylorus so as to prevent the contents of the stomach from passing. Put him upon opium to keep him as easy as possible, as long as life should last. He now took no food, and even water was rejected within a few minutes after taking it.

Some of the friends were not satisfied, and so sent for Dr. L., an old gray-headed man, a former Regular, but now a convert to "similia similibus." He came in my absence. Said the young doctors were all wrong, that the trouble was with the liver, that there was an abscess, which pressing upon the stomach, caused all the trouble. That it was too late now, but if he had seen him in time *he* could have cured him.

For twenty-five days the patient took no food, and had no movement of the bowels. Died at midnight, June 14, of sheer starvation. There was so much dissatisfaction among the relatives, that a *post mortem* was decided upon. I made the examination eighteen hours after death. Our aged homœopathic friend was present. Found the liver perfectly healthy; bowels empty, except a little gas; stomach contained two or three ounces of water, drank just before he died. There was no *diseased* appearance about anything; but partially surrounding the pylorus, and an inch or two of the duodenum, was an irregular-shaped tumor, of perhaps four to six ounces weight, which in a recumbent posture completely closed the outlet of the stomach. The tumor was of a grayish-white color, and appeared to be fibro-cartilaginous, and looked like a perfectly healthy abnormal growth.

